# Clinical trial eligibility of a real-world connective tissue disease cohort: Results from the LEAP cohort

**DOI:** 10.1016/j.semarthrit.2024.152463

**Published:** 2024-08

**Authors:** Sarah Dyball, Anastasia-Vasiliki Madenidou, Mia Rodziewicz, John A. Reynolds, Ariane L. Herrick, Sahena Haque, Hector Chinoy, Ellen Bruce, Ian N. Bruce, Ben Parker

**Affiliations:** a Centre for Musculoskeletal Research, Division of Musculoskeletal and Dermatological Sciences, Faculty of Biology, Medicine and Health, University of Manchester, Manchester Academic Health Science Centre, Stopford Building, Oxford Road, Manchester, UK; b The Kellgren Centre for Rheumatology, Manchester University Hospitals NHS Foundation Trust, Manchester, UK; c Rheumatology Research Group, Institute of Inflammation and Ageing, College of Medical and Dental Sciences, University of Birmingham, Birmingham, UK; d Rheumatology Department, Sandwell and West Birmingham NHS Trust, Birmingham, UK; e National Institute for Health Research Manchester Biomedical Research Centre, Manchester University NHS Foundation Trust, The University of Manchester, Manchester, UK; f Department of Rheumatology, Salford Care Organisation, Northern Care Alliance NHS Foundation Trust, Salford, UK; g Department of Rheumatology, Manchester University Foundation Trust, Wythenshawe Hospital, Manchester, UK

**Keywords:** Connective tissue disease, Lupus, Classification criteria, Overlap syndromes, Clinical trials, Myositis, Systemic sclerosis

## Abstract

**Introduction:**

Classification criteria aim to identify a homogenous population of patients for research. We aimed to quantify how well phase-III trials in connective tissue diseases (CTDs) represent a real-world cohort.

**Methods:**

A comprehensive review of all major published phase-III trials in CTDs was performed (clinicaltrials.gov). Classification criteria utilised most commonly in clinical trials were applied to a multicentre unselected CTD cohort.

**Results:**

There were 42 CTD trials identified, with no trials in mixed (MCTD) or undifferentiated CTD (UCTD). The majority of trials (*N* = 38, 90 %) required patients to meet classification criteria for their respective disease. Eight (19.0 %) excluded patients with overlapping CTDs and a further two (4.8 %) excluded specific overlapping features, such as pulmonary arterial hypertension. One study explicitly allowed overlap syndromes. Our real-world CTD cohort included 391 patients. Patients with UCTD or MCTD (91/391, 23.3 %) would be excluded from participation in clinical trials for not having an eligible diagnosis. Of patients with primary Sjögren's syndrome (pSS), SLE, systemic sclerosis (SSc) or idiopathic inflammatory myopathy (IIM), 211/300 (70.3 %) met the classification criteria for their respective diagnosis and 24/211 (11.4 %) met criteria for >1 CTD. In total, 187/391 (47.8 %) would be eligible for recruitment, based upon their physician diagnosis, and most stringent trial eligibility criteria.

**Conclusion:**

In an unselected, real-world CTD cohort, up to half of patients are ineligible for clinical trials due to not meeting classification criteria, overlapping features or a lack of trials within their primary disease. To address this inequality in access to novel therapies, clinical trial design should evolve eligibility criteria in CTDs.

## Highlights

• Significant proportion of CTD patients are excluded from phase-III clinical trials.
• Classification criteria may take several years to be adopted into trial protocols.
• Stringent trial eligibility may lead poor generalisability for real-world patients.

## Graphical abstract

**Figure:**
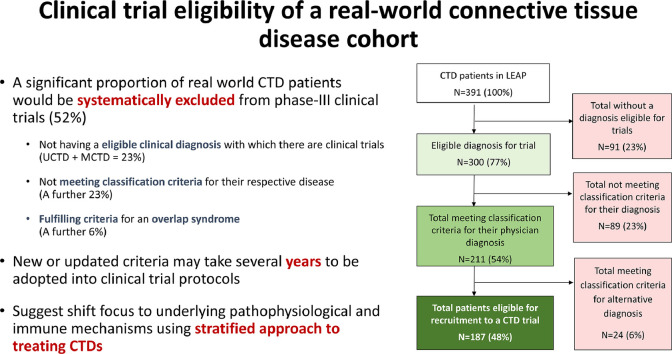

## Introduction

Connective tissue diseases (CTDs), also known as systemic autoimmune rheumatic diseases, are a complex and broad group of multisystem diseases with a heterogeneous presentation and diverse clinical course. Diagnosis is made on an individualised basis by specialist evaluation of clinical manifestations alongside supportive investigations. Diagnosis remains a challenge, in part due to similarities across diseases and ill-defined phenotypes, particularly during the early stages of disease [1,2].

Previous work from our group has shown that in SLE, two-thirds of patients would be excluded from participating in phase-III clinical trials due to exclusion criteria (such as prohibited medications use, trials mandating a severity level of disease activity, and presence of comorbidities) and for not meeting classification criteria [3]. Classification criteria have standardised definitions, which aim to include key features of disease to identify a homogenous population of patients with a high specificity for research [4,5]. Given that CTDs, beyond lupus, are associated with significant morbidity, mortality and impairment in health-related quality of life [6,7], clinical trials must be generalisable to our patient-population for them to be useful.

This study builds on our previous study by determining how phase-III trials across the spectrum of CTDs utilise classification criteria within their inclusion and exclusion criteria. We have applied the most commonly used criteria to a large unselected cohort of patients with an existing CTD diagnosis to explore which patients would be included and excluded from clinical trials.

## Methods

### Identification of eligible trials on "ClinicalTrials.gov"

We searched “ClinicalTrials.gov” database which records privately and publicly funded clinical studies conducted around the world on 11th November 2022. To identify relevant studies, we used the search terms “lupus” or “systemic lupus erythematosus”, “myositis” or “inflammatory myopathy” or “inflammatory muscle disease” or “muscle inflammation”, “systemic sclerosis” or “scleroderma”, “UCTD” or “undifferentiated connective tissue disease”, “MCTD” or “mixed connective tissue disease” and “Sjögren's”. We aimed for the clinical trials included in the review to capture trials used in the licencing of DMARDs and biologics in patients with CTDs. We therefore included interventional phase-III clinical trials involving CTD patients, who were treated with either a biologic therapy or DMARD and excluded trials which included non-inflammatory diseases, long term extensions of previously published RCTs, open label studies, or studies not deemed to be the principal study, such as those using an alternative method of administration e.g. BLISS-SC (NCT01484496).

Two reviewers (SD and AM) independently reviewed the studies, and procured full manuscripts and trial protocols where available. Data extraction was performed independently (SD and AM) using a standardised form; disagreement was resolved by consensus.

### Patients and study design of the leap cohort

The Lupus Extended Autoimmune Phenotype (LEAP) cohort is a prospective multicentre study of patients with a diagnosed CTD. From May 2014 - September 2022, adult patients were recruited from five UK National Health Service (NHS) rheumatology departments into the cohort. The LEAP cohort includes patients with SLE, primary Sjögren's syndrome (pSS), UCTD, systemic sclerosis (SSc), mixed connective tissue disease (MCTD) and idiopathic inflammatory myopathy (IIM).

Patients with an established diagnosis and clinically stable disease were eligible for inclusion if they had ≥ 1 clinical feature of a CTD and ≥ 1 positive autoantibody ever reported within the antinuclear antibody (ANA) spectrum. A full list of manifestations is recorded within the supplemental appendix. Rheumatologist diagnosis at the time of recruitment was used as the primary classifier of patients, and patients were not required to meet classification criteria. As such, the group of patients with MCTD may have included those with overlapping clinical features. UCTD was defined as clinical and laboratory findings typical for CTD but not fulfilling the classification criteria for a definite CTD. All patients signed written informed consent and the study was conducted in accordance with the Declaration of Helsinki (Ethical approval: 13/NW/0564).

### Classification criteria from trials applied to the leap cohort

Following the identification of eligible trials, we applied the most commonly used classification criteria in the reviewed clinical trials to the LEAP cohort. These criteria included the American College of Rheumatology (ACR) 1997 SLE [8], Bohan and Peter criteria for myositis (probable or definite diagnosis of dermatomyositis [DM] or polymyositis [PM]) [9,10], the ACR 1980 criteria for SSc [11] and the 2002 American-European Consensus Group criteria (AECG) criteria for pSS [12]. The classification criteria utilised in this research are summarised in Supplemental Tables S1–4. In order to review whether newer classification criteria were more inclusive of CTD patients, a secondary analysis was completed whereby the most recent classification criteria for each disease were also applied to this cohort. These included the European Alliance of Associations for Rheumatology (EULAR)/ ACR-2019 criteria for SLE [4], the 2017 EULAR/ACR criteria for IIM [13], the 2013 ACR/EULAR criteria for SSc [4] and the 2016 EULAR/ACR criteria for pSS [14]. EULAR Sjögren's syndrome disease activity index (ESSDAI) scores were not collected, therefore, all patients classified as pSS using 2016 EULAR/ACR criteria for pSS had to report symptoms of dryness. The results of muscle biopsies were not available for all patients, therefore, when calculating the 2017 EULAR/ACR IIM criteria, clinical criteria were used.

### Statistical analysis

Baseline demographic data are presented using descriptive statistics performed using R (V4.2.1) and Venn diagrams were created using the package limma (V3.28.14). Differences between groups were analysed using Mann-Whitney U test for continuous data and Chi-Squared test for categorical data. A logistic regression model was used to test the age, sex and clinician diagnosis adjusted association between trial eligibility and previous rheumatic therapy use.

## Results

### Identification of clinical trials

#### Clinical trial characteristics

The comprehensive literature review, using clinicialtrials.gov, identified *n* = 1916 trials; of these, 1793 were excluded at abstract screening, and 81 at full-text screening. The study selection process, as per PRISMA guidelines, is shown in Supplemental Figure S5, with our final analysis including 42 studies. This included 20 studies in SLE, 12 in SSc, six in pSS and four in IIM. There were no studies in MCTD or UCTD. Included trials and their inclusion and exclusion criteria are shown in Supplemental Table S6.

#### Inclusion criteria in sle trials

Twenty studies in SLE and lupus nephritis were included. Nineteen SLE studies required patients to meet the ACR criteria for SLE of which 14 specified the revised 1997 criteria [8], and four the 1982 criteria [15]. One study specified a clinical diagnosis of SLE (NCT00470522, methotrexate) which was the oldest SLE trial included.

Two studies (NCT02446912; NCT02446899, anifrolumab) excluded patients with a diagnosis of MCTD within a year, or any history of overlap syndromes of SLE and SSc. The AURORA (NCT03021499, voclosporin) trial in lupus nephritis excluded any overlapping autoimmune condition for which the condition or the treatment of the condition may affect the study assessments or outcomes (e.g. scleroderma with significant pulmonary arterial hypertension; any condition for which additional immunosuppression is indicated), however overlapping conditions for which the condition or treatment was not expected to affect assessments or outcomes (e.g. Sjögren's syndrome) were not excluded. Eleven studies excluded patients with concomitant medical conditions which may interfere with their safety or the evaluation of the study drug, as determined by the investigator. Six studies made no reference to overlap syndromes.

#### Inclusion criteria in pSS trials

There were six trials included in pSS. Five studies required patients to meet the AECG criteria for pSS 2002 [12], and one study (NCT02915159, abatacept) required patients to meet the 2016 ACR/EULAR criteria for pSS [14]. Five studies excluded secondary Sjögren's syndrome. One study (NCT01601028, hydroxychloroquine) did not explicitly exclude patients with secondary Sjögren's or other autoimmune diseases.

#### Inclusion criteria in SSc trials

There were twelve trials included in SSc, of which nine required patients to meet specific classification criteria. For four this was the ACR definition of scleroderma 1980, and for three the ACR-EULAR 2013 criteria for SSc [5,11]. One trial (NCT01570764, cyclophosphamide) required patients to meet either the ACR definition of scleroderma 1980 or the LeRoy and Medsger 2001 criteria for early SSc [16], and one trial (NCT01748084, rituximab) the ACR definition of scleroderma 1980 or the LeRoy 1988 classification criteria [17]. Only the three oldest trials enrolled patients with a clinical diagnosis of systemic sclerosis; two trials required a clinical diagnosis of diffuse SSc ((NCT00704665, relaxin; NCT00070590, bosentan), and one diffuse or limited SSc (NCT00348296, Venoglobulin-IH). Five studies excluded patients with a rheumatic autoimmune disease other than SSc. One study investigating interstitial lung disease (ILD) in SSc (NCT00070590, bosentan) excluded ILD due to any other condition other than SSc, and six studies made no comment.

#### Inclusion criteria in iim trials

There were four trials of patients with IIM, two of DM and two for PM or DM. All studies required patients to meet classification criteria for myositis; three Bohan and Peter criteria, one Bohan and Peter or the EULAR/ACR 2017 criteria for DM [9,10,13]. One study (NCT02728752, Octagam 10 %) explicitly excluded patients with diagnoses other than primary idiopathic PM or DM, such as drug-induced myositis, myositis in association with other CTD (except Sjögren's), inclusion body myositis, malignancy related myositis, and juvenile DM. One study allowed overlap with features of SSc, SLE, Sjögren's syndrome or rheumatoid arthritis if the dominant clinical disease was DM (NCT03813160, lenabasum). The two other studies made no reference to excluding overlap syndromes (NCT00335985, GB-0998; NCT01165008, anakinra).

### The leap cohort

#### Patient demographics of the leap cohort

Data were collected from 391 patients (352 [90.0 %] women, with a median [IQR] age of 52 [40–59] years), described in table 1. By rheumatologist diagnosis, 164 patients (41.9 %) had SLE, 77 (19.7 %) pSS, 61 (15.6 %) UCTD, 37 (9.5 %) SSc, 22 (5.6 %) IIM and 30 (7.7 %) MCTD. Patients with SSc, MCTD or pSS were older than those with SLE. Disease duration differed across diagnostic groups (kwallis, *p* < 0.001), with the longest disease duration being in patients with SLE (median [IQR] 9.9 [3.9–17.2] years).

**Table 1:** demographics, therapeutics and disease manifestations across CTD diagnoses from an unselected CTD cohort (LEAP cohort). Renal involvement was defined as persistent proteinuria >0.5 g per day or >3+ on urine dipstick testing or renal tubular acidosis attributable to CTD, or scleroderma renal crisis. Data reported as median (IQR) or N (%) as appropriate. Anti-dsDNA, anti-double stranded DNA; anti-RNP, anti-ribonucleoprotein antibodies; IIM, idiopathic inflammatory myopathies; MCTD, mixed connective tissue disease; pSS, primary Sjögren's syndrome; SSc, systemic sclerosis; UCTD, undifferentiated connective tissue disease.

	SLE	pSS	UCTD	SSc	MCTD	IIM	Overall
	(*N* = 164)	(*N* = 77)	(*N* = 61)	(*N* = 37)	(*N* = 30)	(*N* = 22)	(*N* = 391)
**Demographics**							
Gender							
Female	150 (91.5)	74 (96.1)	52 (85.2)	32 (86.5)	25 (83.3)	19 (86.4)	352 (90.0)
Male	14 (8.5)	3 (3.9)	9 (14.8)	5 (13.5)	5 (16.7)	3 (13.6)	39 (10.0)
Age (years)	47.0 (34.0–54.3)	56.0 (46.0–61.0)	49.0 (36.0–56.0)	60.0 (57.0–66.0)	50.0 (40.0–54.8)	56.5 (52.5–61.8)	52.0 (40.0–59.0)
Disease duration (years)	9.9 (3.9–17.2)	4.60 (2.9–8.1)	4.30 (2.1–6.6)	7.0 (3.4–14.8)	7.0 (4.8–15.9)	3.3 (1.7–6.7)	6.1 (2.9–13.2)
Ethnicity							
White	122 (74.4)	62 (80.5)	38 (62.3)	31 (83.8)	23 (76.7)	17 (77.3)	293 (74.9)
Asian	13 (7.9)	6 (7.8)	2 (3.3)	4 (10.8)	2 (6.7)	0 (0)	27 (6.9)
Black	23 (14.0)	4 (5.2)	13 (21.3)	1 (2.7)	5 (16.7)	3 (13.6)	49 (12.5)
Other	6 (3.7)	5 (6.5)	8 (13.1)	1 (2.7)	0 (0)	2 (9.1)	22 (5.6)
**Prior therapeutics**							
Oral steroids	117 (71.3)	24 (31.2)	20 (32.8)	13 (35.1)	16 (53.3)	15 (68.2)	205 (52.4)
Immunosuppressants	88 (53.7)	22 (28.6)	14 (23.0)	8 (21.6)	21 (70.0)	15 (68.2)	168 (43.0)
Biologics	15 (9.1)	2 (2.6)	3 (4.9)	2 (5.4)	3 (10.0)	2 (9.1)	27 (6.9)
**Manifestations**							
Sicca	53 (32.3)	75 (97.4)	17 (27.9)	12 (32.4)	9 (30.0)	5 (22.7)	171 (43.7)
Inflammatory arthritis	87 (53.0)	25 (32.5)	16 (26.7)	8 (21.6)	21 (70.0)	6 (28.6)	163 (42.0)
Oral ulcers	85 (51.8)	16 (20.8)	11 (18.0)	3 (8.1)	6 (20.0)	1 (4.5)	122 (31.2)
Photosensitivity	90 (54.9)	22 (28.6)	14 (23.0)	4 (10.8)	8 (26.7)	7 (31.8)	145 (37.1)
Raynaud's phenomenon	85 (52.5)	29 (38.2)	29 (49.2)	37 (100)	23 (76.7)	10 (45.5)	213 (55.2)
Renal involvement	36 (22.0)	0 (0)	0 (0)	1 (2.7)	4 (13.3)	0 (0)	41 (10.5)
Anti-RNP	42 (25.6)	5 (6.5)	9 (14.8)	2 (5.4)	23 (76.7)	0 (0)	81 (20.7)
Anti-Ro	48 (29.3)	51 (66.2)	16 (26.2)	4 (10.8)	6 (20.0)	5 (22.7)	130 (33.2)
Anti-Smith	28 (17.1)	2 (2.6)	2 (3.3)	1 (2.7)	12 (40.0)	0	45 (11.5)
Anti-La	25 (15.2)	32 (41.6)	9 (14.8)	0	0	1 (4.5)	67 (17.1)
Anti-dsDNA	71 (43.3)	10 (13.0)	9 (14.8)	1 (2.7)	3 (10.0)	1 (4.5)	95 (24.3)
Anti-topomerase I	6 (3.7)	1 (1.3)	3 (4.9)	4 (10.8)	2 (6.7)	0	16 (4.1)
Anti-centromere	3 (1.8)	0	1 (1.6)	17 (45.9)	2 (6.7)	0	23 (5.9)
Anti-Jo1	2 (1.2)	1 (1.3)	1 (1.6)	0	0	7 (31.8)	11 (2.8)

#### Disease manifestations and therapeutics across the leap cohort

Certain CTD manifestations, including both clinical features (e.g. cytopenias, and inflammatory arthritis) and autoantibody profiles (e.g. anti-Ro and anti-RNP antibody) were associated with every disease group in the cohort. Raynaud's phenomenon was present in 213/386 (55.2 %) of the cohort, most commonly in SSc (37/37, 100 %) and MCTD (23/30, 76.7 %) patients. Subjective sicca symptoms were present in 171/391 (43.7 %) of the cohort. Inflammatory arthritis was seen in 163/388 (42.0 %) of patients, most commonly in MCTD (21/30, 70.0 %) and SLE (87/164, 53.0 %). A photosensitive rash was reported in 37.1 % of the cohort. Anti-Ro antibody was the most commonly exhibited autoantibody (130/383, 33.2 %).

Prior therapeutic strategies were reviewed across diseases, and a proportion of patients from each CTD diagnosis prescribed oral corticosteroids, immunosuppressants and biologics. Previous oral steroid use was highest in SLE and IIM (*n* = 117/164, 71.3 % and *n* = 15/22, 68.2 %, respectively) and immunosuppressant use was highest in IIM (*n* = 15, 68.2 %), MCTD (*n* = 21, 70.0 %) and SLE (*n* = 88, 53.7 %) patients.

#### Diseases eligible for recruitment to clinical trials

**Fig. 1:**
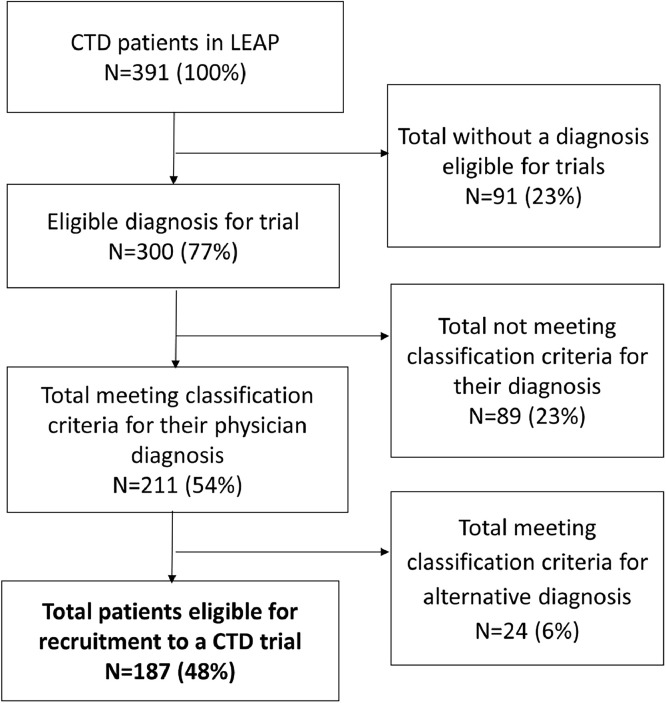
the inclusion and exclusion criteria from phase-III clinical trials applied to an unselected CTD cohort (LEAP cohort); CTD, connective tissue diseases.

Ninety-one (23.3 %) patients had UCTD or MCTD for which there were no RCTs and would therefore not be eligible for clinical trial recruitment, Fig. 1.

#### Patients meeting classification criteria for their respective diagnosis

211/300 (70.3 %) patients with pSS, SLE, SSc or IIM, met the most commonly utilised classification criteria for their respective diagnosis, (Fig. 1& table 2). This was highest in patients with SLE (*N* = 138, 84.2 %) and pSS (*N* = 45, 58.4 %), and lowest in SSc (*N* = 19, 51.4 %) and IIM (*N* = 9, 40.9 %).

**Table 2:** classification criteria used most commonly in Phase-III clinical trials applied to a CTD unselected cohort, stratified by their rheumatologist made diagnosis. ACR, American college for Rheumatology; AECG American-European Consensus Criteria; IIM, idiopathic inflammatory myopathies; MCTD, mixed connective tissue disease; pSS, primary Sjögren's syndrome; SSc, systemic sclerosis; UCTD, undifferentiated connective tissue disease.

	SLE	pSS	UCTD	SSc	MCTD	IIM	Overall
	*N* = 164	*N* = 77	*N* = 61	*N* = 37	*N* = 30	*N* = 22	*N* = 391
ACR SLE 1997	138 (84.1)	14 (18.2)	0	2 (5.4)	14 (46.7)	5 (22.7)	173 (44.2)
AECG Sjögren's	10 (6.1)	45 (58.4)	0	0	1 (3.3)	1 (4.5)	57 (14.6)
ACR Systemic sclerosis 1980	0	0	0	19 (51.4)	5 (16.7)	4 (18.2)	28 (7.2)
Bohan and Peter for IIM	2 (1.2)	0	0	3 (8.1)	2 (6.7)	9 (40.9)	16 (4.1)

#### Patients meeting classification criteria outside of their diagnosis

Of the patients with an eligible diagnosis and who met their respective classification criteria, 24/211 (11.4 %) would be excluded from clinical trials for meeting the classification criteria for an alternative CTD diagnosis (Fig. 1).

**Fig. 2:**
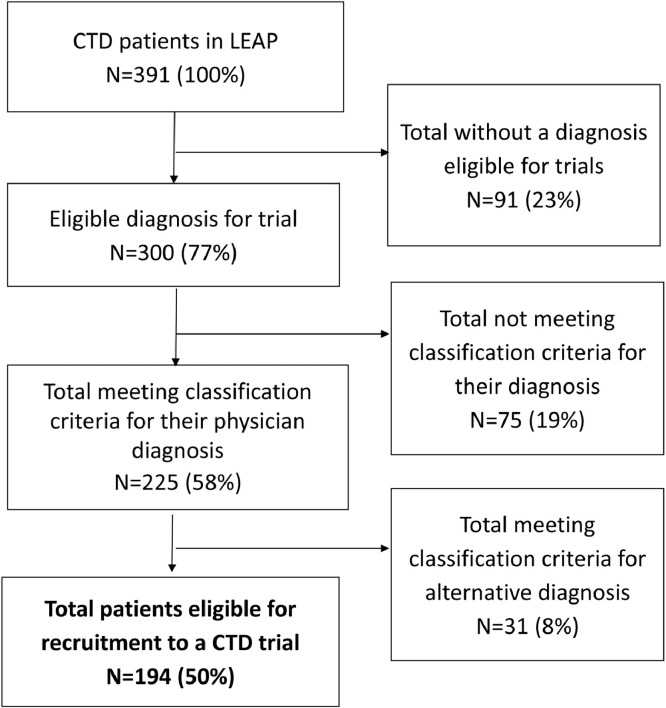
the inclusion and exclusion criteria from phase-III clinical trials applied to an unselected CTD cohort, using most recent iterations of classification criteria.

Across the whole cohort, 243 (62.1 %) patients met classification criteria for at least one CTD, and 31 (7.9 %) met the criteria for two CTDs. No patient met classification criteria for three or more CTDs. The most common overlap included patients meeting the criteria for SLE and pSS (16/391, 4.1 %) of whom nine patients had a diagnosis of pSS, and seven had a diagnosis of SLE. This was followed by SLE and SSc (8/391, 2.0 %); of whom two had a diagnosis of SSc, six had IIM and four had a diagnosis of MCTD. Almost all MCTD patients (90 %) met classification criteria for an alternative CTD (figure S7) which would be a leading indication for clinical trial inclusion. Furthermore, 22.7 % IIM and 6.5 % of pSS patients met classification criteria for an alternative diagnosis without meeting classification criteria for their primary diagnosis. The full spectrum of overlaps is shown in Fig. 2, and by clinician diagnosis in Supplemental Figures S7–12.

No patients with UCTD met any of the classification criteria most utilised in our review of clinical trials. Of the 30 patients with MCTD, 17 (56.6 %) patients met at least one classification criteria; 14 (46.7 %) patients met ACR-1997 criteria for SLE, 5 (16.7 %) the 1980 ACR criteria for SSc, 2 (6.7 %) the Bohan and Peter criteria for IIM, and 1 (3.3 %) met the AECG criteria for pSS. Five (16.7 %) MCTD patients met the criteria for more than one CTD.

#### Eligibility of patients by clinician diagnosis

Patients with SLE were the most likely to be eligible for recruitment in clinical trials (130/164, 79.3 %) in terms of meeting classification criteria, and not fulfilling criteria for an overlap condition. This was followed by pSS (36/77, 46.8 %), SSc 15/37 (40.5 %) and IIM (6/22, 27.3 %). No patients with UCTD or MCTD were eligible.

#### Characteristics and differences of patients not meeting eligibility criteria

Compared to the 204 (52 %) CTD patients not eligible for recruitment to clinical trials in this cohort, eligible patients were younger in age (OR 0.98 [0.97–1.00]) and had a shorter disease duration (OR 1.03 [1.00–1.05]). When adjusted for differences in age, gender and diagnostic group, there were no significant difference in previous medication use including steroids (OR 0.57 [0.32–1.05]), DMARDs (OR 1.50 [0.86–2.62]) and biological therapies (OR 0.92 [0.32–2.63]) between those eligible and ineligible.

#### Application of most recent iteration of classification criteria

**Fig. 3:**
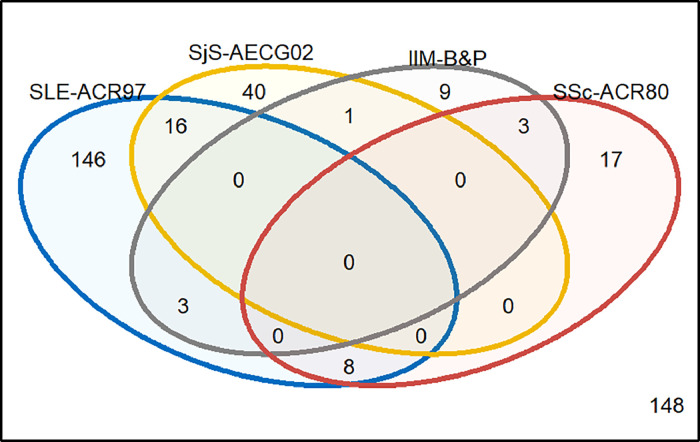
Venn diagram showing classification criteria most commonly used in clinical trials applied to 391 patients from LEAP, an unselected CTD cohort. The circles represent patients meeting the classification criteria for the American College of Rheumatology (ACR) 1997 SLE (blue), the 2002 American-European Consensus Group criteria (AECG) criteria for pSS (yellow), Bohan and Peter criteria for myositis (probable or definite diagnosis of dermatomyositis [DM] or polymyositis [PM]) (grey), and the ACR 1980 criteria for SSc (red). Numbers in intersecting circles represent the number of patients meeting the classification criteria for multiple criteria. There were 148 patients who did not meet any classification criteria.

Three trials of SSc (NCT02597933, nintedanib; NCT02453256, tocilizumab; NCT04274257, rituximab), one of IIM (NCT03813160, lenabasum), one of pSS (NCT02915159, abatacept) and no study of SLE patients used the most recent classification criteria (ACR/ EULAR 2019 criteria for SLE, ACR/EULAR 2017 criteria for IIM, ACR/EULAR 2016 criteria for pSS, ACR/ EULAR 2013 criteria for SSc). Application of these criteria to our cohort increased the number of patients meeting the criteria for their respective disease except in SLE, as shown in Table 3. 225 (75.0 %) patients with pSS, SLE, SSc or IIM, met the classification criteria for their respective diagnosis, (Fig. 3& Table 3). This was highest in patients with SSc (*N* = 32, 86.5 %) and SLE (*N* = 128, 78.1 %), and lowest in IIM (*N* = 15, 68.2 %) and pSS (*N* = 50, 64.9 %), of these patients, 28/225 (12.4 %) met criteria for >1 CTD. The most common overlap was patients meeting the criteria for SLE and pSS (17, 4.3 %). In total, using the most recent iterations of classification criteria, 194/391 (49.6 %) would be eligible, and 197/391 (50.4 %) ineligible for recruitment to a phase-III trial.

**Table 3:** most recent iterations of classification criteria applied to a CTD unselected cohort, stratified by their rheumatologist made diagnosis. ACR, American college for Rheumatology; EULAR, European Alliance of Associations for Rheumatology; IIM, idiopathic inflammatory myopathies; MCTD, mixed connective tissue disease; pSS, primary Sjögren's syndrome; SSc, systemic sclerosis; UCTD, undifferentiated connective tissue disease.

	SLE	pSS	UCTD	SSc	MCTD	IIM	Overall
	*N* = 164	*N* = 77	*N* = 61	*N* = 37	*N* = 30	*N* = 22	*N* = 391
EULAR/ACR SLE 2019	128 (78.1)	16 (20.8)	8 (13.1)	2 (5.4)	17 (56.7)	1 (4.6)	172 (44.0)
ACR/EULAR Sjögren's 2016	12 (7.3)	50 (64.9)	0 (0)	1 (2.7)	2 (6.7)	1 (4.6)	66 (16.9)
ACR/EULAR Systemic sclerosis 2013	1 (0.6)	0	0	32 (86.5)	8 (26.7)	4 (18.2)	45 (11.5)
EULAR/ACR 2017 for IIM	4 (2.4)	1 (1.3)	1 (1.6)	3 (8.1)	0	15 (68.2)	24 (6.1)

## Discussion

Our study found that 52 % of patients from an unselected CTD cohort would not be eligible for recruitment to phase-III clinical trials based on their clinical diagnosis and most commonly used clinical trial eligibility criteria. There were no phase-III trials in UCTD and MCTD. Largely, clinical trials (38/42, 90 %) required patients to meet the classification criteria for their respective diagnosis, with only the oldest clinical trials in SLE and SSc using clinical diagnosis. Clinical trials in pSS and to a lesser extent in SSc, required patients not to have a co-existing CTD whereas this was less stringent in SLE and IIM trials. Finally, the classification criteria used in most clinical trials were commonly not the most recent iteration and were less sensitive, with it taking several years for new classification criteria to be adopted into clinical trial protocols.

Clinical trial recruitment is challenging, in part due to strict inclusion criteria which is based upon meeting classification criteria. However, this is only one aspect of trial eligibility, therefore beyond the 52 % of patients in this study not fulfilling these criteria, this number will be further increased due to additional eligibility criteria including prohibited medications use, trials mandating a severity level of disease activity and comorbidities such as chronic kidney disease or previous malignancies.

The requirement of patients to meet classification criteria results in patients with rare manifestations (e.g. chorea or transverse myelitis), or those with common but non-specific manifestations that lack specificity (e.g. Raynaud's phenomenon, arthralgia, inflammatory rashes) from being excluded from trials. A significant number of patients did not meet the criteria for their respective disease, most notably in IIM and SSc. This improved with newer classification criteria, notably the 2013 ACR/EULAR criteria for SSc, which has a higher sensitivity for limited cutaneous forms of disease [5]. This study highlights how new or updated criteria may take several years to be adopted into clinical trial protocols. Despite this,limiting recruitment to only those meeting clinically-based classification criteria narrows the recruitment pool for selecting patients who may benefit from new medications, as well as excluding patients from enrolment into clinical trials where participation is associated with improved outcomes [18]. We know that patients ‘excluded’ by classification criteria still have a high burden of disease and high damage accrual [19], but there are many barriers to include these patients in trials despite this unmet need.

Patients with SLE were most likely to be eligible for recruitment to clinical trials. This may relate to inherent characteristics of the SLE classification criteria. Further, patients with a diagnosis of SLE had a longer disease duration compared with patients with other diagnoses, meaning they had longer to meet criteria for their disease. Interesting, patients met classification criteria for diseases outside of their primary, physician-made diagnosis, most notably for the ACR97 SLE criteria. Notably, some patients met classification criteria for alternative CTDs, without meeting criteria for their primary diagnosis. If classification criteria were used to define a diagnosis, a group of patients could enrol in trials with an alternative leading diagnosis.

Both UCTD and MCTD remain under-represented in clinical research, with no phase-III clinical trials to date and no licenced disease-modifying therapies. This study highlights the unmet need for patients with UCTD and MCTD where treatment strategies must be repurposed from other conditions [20,21]. Many disease manifestations cross clinical disease boundaries, and the impact of any novel therapy could be underestimated using trial methodology based purely on clinical criteria. Basket trials are a novel trial design in which targeted therapies are evaluated across multiple diseases which have common molecular alterations [22]. To our knowledge, MCTD has only been included in clinical trials which utilise a basket-trial design [23].

The main purpose of classification criteria is to ensure a homogeneous cohort of patients to enrol in all research studies. Ensuring some degree of similarity between patients is important to allow comparison of different patient cohorts. As such, classification criteria emphasise specificity to avoid false positives. However, by definition, CTDs are heterogeneous both across and within diseases. When assessing the strengths and limitations of each set of classification criteria, the most pertinent question is for what purpose they are being used. Results from our unselected CTD cohort show that certain disease manifestations (e.g. Raynaud's phenomenon, cytopenias, and inflammatory arthritis) and autoantibody profiles (e.g. anti-Ro and anti-RNP) span every CTD group. The therapeutics presently used in the management of these conditions also cross diseases, with a proportion of patients from each group using immunosuppressant, steroids and biological therapies. We have previously shown that raised interferon stimulated gene (ISG) scores can be seen across the spectrum of CTDs, with these correlating to specific clinical features and autoantibody profiles [24]. The PRECISESADS project has used ‘omics and bioinformatics to identify new classifications for CTDs based on shared pathophysiological mechanisms in view of personalised treatments [25]. Commonalities in symptoms, therapeutics and molecular signatures shows that a basket trial approach in CTDs should be achievable. We would argue for a paradigm shift in clinical trial design that moves away from relying on classification criteria to define the disease, and instead, uses a stratified medicine approach to define the molecular taxonomy of CTDs. This would allow patients with diagnoses outside of current clinical trials (e.g. UCTD and MCTD) and those not meeting the classification criteria for their respective CTD to be included within clinical trials.

A potential weakness of the study is that our gold standard was a diagnosis made by a specialist rheumatologist at recruitment to the LEAP study. Given the inherent problems involved in developing diagnostic criteria in such a complex disease area, clinician diagnosis remains the most accurate means of diagnosing such diseases [26]. A further limitation is that the LEAP study was designed and initiated prior to the publication of the EULAR-ACR19 criteria for SLE, the EULAR-ACR16 criteria for pSS and the EULAR-ACR17 criteria for IIM. Data were therefore not collected on all aspects of disease manifestations, including whether pericarditis was confirmed by objective evidence. Entry criterion for pSS criteria mandate that patients have either symptoms of ocular or oral dryness or an ESSDAI score ≥1. In this cohort, ESSDAI scores were not collected, therefore, all patients classified as pSS had to report symptoms of dryness. The results of muscle biopsies were not available for all patients, therefore, when calculating the 2017 EULAR/ACR IIM criteria in these patients, we assumed that this was not present. Employing this methodology may have led to small changes in the number of patients who were eligible for clinical trial participation in the sensitivity analysis.

## Conclusions

In summary, just over half of patients in a real world CTD cohort would be systematically excluded from phase-III clinical trials due to the requirement for patients to meet classification criteria for their respective disease, having a diagnosis with which there are no clinical trials, or for fulfilling criteria for an overlap syndrome. Furthermore, new or updated classification criteria may take several years to be adopted into clinical trial protocols. Clinical trials design should reconsider eligibility and exclusion criteria to be more inclusive and thus ensure the generalisability of clinical trials.

## CRediT authorship contribution statement

**Sarah Dyball:** Conceptualization, Methodology, Formal analysis, Writing – original draft, Writing – review & editing. **Anastasia-Vasiliki Madenidou:** Formal analysis, Writing – review & editing. **Mia Rodziewicz:** Formal analysis, Writing – review & editing. **John A. Reynolds:** Resources, Writing – review & editing. **Ariane L. Herrick:** Resources, Writing – review & editing. **Sahena Haque:** Resources, Writing – review & editing. **Hector Chinoy:** Resources, Writing – review & editing. **Ellen Bruce:** Resources, Writing – review & editing. **Ian N. Bruce:** Conceptualization, Writing – review & editing, Supervision, Funding acquisition. **Ben Parker:** Conceptualization, Writing – review & editing, Supervision, Funding acquisition.

## Declaration of competing interest

S.D. has received grant support from Novartis and UCB. A.M. has received grant support from Janssen and UCB. MR has received grant support from UCB. A.L.H. has received grant support from Gesynta, consultancy fees from Arena, Boehringer-Ingelheim, Camurus, CSL-Behring, Galderma and Gesynta, and speaker fees from Janssen, H.C. has received grant support from Eli Lilly and UCB; consulting fees from Novartis, Eli Lilly, Orphazyme, Astra Zeneca; speaker for UCB, Biogen. I.N.B. has received grant support from Genzyme/Sanofi, GSK, Roche and UCB; consulting fees from AstraZeneca, Eli Lilly, GSK, Merck Serono, UCB and ILTOO; and was a speaker for AstraZeneca, GSK and UCB. BP has received grant support from Genzyme/Sanofi and GSK, honoraria from Fresenius-Kabi and AbbVie and was a speaker for Eli Lilly and Roche.

## Appendix Supplementary materials

Image, application 1
