# Supplementary material for: Clinical trial eligibility of a real-world connective tissue disease cohort: Results from the LEAP cohort
Source: Semin Arthritis Rheum. 2024 Aug;67:None. doi: 10.1016/j.semarthrit.2024.152463 (PMC11199160; doi:10.1016/j.semarthrit.2024.152463)
Supplement: Supplementary file 1 [file mmc1.docx]

**List of manifestations collected in LEAP**

Has the participant ever had or do they currently have any of the following **attributable to their CTD?**

**Mucocutaneous features**

Symptomatic dry mouth

Objective dry mouth

Panniculitis

Major cutaneous vasculitis/thrombosis

Cutaneous vasculitis

Alopecia

Chillblains

Discoid rash

Urticarial vasculitis

Oral ulcers (recurrent)

Nasal ulcers (recurrent)

Gottron's papules

Gottron's sign

Digital tip ulcers

Fingertip tipping scars

Skin thickening proximal to MCP

Sclerodactyly (between MCP and PIP)

Raynaud's phenomenon

Puffy fingers

Malar rash

Photosensitivity

DM-specific rash (heliotrope, V sign, shawl sign, poikiloderma)

Heliotroph rash

Calcinosis cutis

Livedo reticularis

Mechanics hands

Subacute cutaneous lupus

Telangiectasia

Lymphocytic sialadentits (on biopsy)

Abnormal nail fold capillaries

**Constitutional**

Pyrexia - documented > 38°C (not due to infection)

Weight loss - unintentional > 5%

Lymphadenopathy

Fatigue/Malaise/Lethargy

**Neuropsychiatric**

Cerebral vasculitis

Myelopathy

Acute confusional state

Psychosis (in the absence of offending drugs/known metabolic derangements)

Acute inflammatory demyelinating

Polyradiculoneuropathy

Neuropathy (cranial, polyneuropathy or mononeutrits multiplex)

Seizures (in the absence of offending drugs/known metabolic derangements)

Cognitive dysfunction

**Musculoskeletal**

Monoarthritis

Oligoarthritis

Polyarthritis <10 joints

Polyarthritis >10 joints

Large joints

Small joints

Erosive arthritis

Tendonitis/Tenosynovitis

Rheumatoid nodules

Proximal muscle weakness

Myogenic changes on electromyography

Muscle biopsy pathology

Tendon contractures

Elevated creatine kinase

**Cardiorespiratory**

Myocarditis

Serositis: lung (Pleurisy or effusion)

Serositis: heart (pericarditis or effusion)

Pulmonary arterial hypertension

Interstitial lung disease

Diffuse alveolar damage

Respiratory bronchitis

Desquamative interstitial pneumonia

Lymphoid interstitial pneumonia

Shrinking lung syndrome

Arterial thrombosis

Venous thrombosis

Dysphonia

**Gastrointestinal**

Recurrent/persistent swollen parotid/salivary glands

Abdominal serositis or ascites

Malabsorption

Autoimmune hepatitis

Primary biliary cirrhosis

Pancreatitis

Oesophageal dysmotility including reflex oesophagitis

Dysphagia

**Ophthalmic**

Symptomatic dry eyes

Objective dry eyes

Orbital inflammation/myositis/proptosis

Keratitis

Uveitis (anterior or posterior)

Episcleritis

Scleritis

Optic neuritis

Corneal ulceration/melt

**Renal**

Accelerated hypertension

Scleroderma renal crisis

Glomerulonephritis

Proteinuria

Granular casts

Renal tubular acidosis

Renal calcification

**Haematological**

Hemolytic Anemia

Leukopenia

Lymphopenia

Thrombocytopenia

Microvascular haemolysis

**Pregnancy morbidity**

1 or more unexplained fetal loss >10 week gestation

1 or more premature births before 34 weeks (eclampsia/pre-eclampsia/placental insufficiency)

3 or more unexplained miscarriages <10 weeks

Classification criteria in primary Sjögren’s Syndrome

|  | **2002 AECG criteria** | **2016 ACR/EULAR criteria** | **Weight** |
| --- | --- | --- | --- |
| Items | 1. Ocular dryness symptoms | 1. Labial salivary gland with focal lymphocytic sialadenitis and focus score ≥1 foci/4mm2 | 3 |
|  | 2. Oral dryness symptoms | 2. Anti-SSA antibodies | 3 |
|  | 3. Ocular signs: Schirmer’s test ≤5 mm/5 min or van Bijsterveld score ≥4 | 3. Ocular staining score ≥5 (or van Bijsterveld score ≥4) in at least one eye | 1 |
|  | 4. Focus score ≥1 foci/4mm2 on minor salivary gland biopsy | Schirmer’s tests ≤5 mm/5min in at least one eye | 1 |
|  | 5. Salivary gland involvement: unstimulated whole salivary flow ≤0.1 ml/ min | Unstimulated whole saliva flow rate ≤0.1ml/min | 1 |
|  | 6. Positive anti-SSA or anti-SSB antibodies |  |  |
| Rules for classification | Absence of exclusion criteria | Applies to any individual who |  |
|  | Presence of any 4 or the 6 items with at least items 4 or 6 | Meets the inclusion criteria with at least one symptom of ocular or oral dryness or ESSDAI ≥1 |  |
|  | Or, presence of any 3 of the 4 objective items (3, 4, 5, and 6) | Does not have any of the conditions listed as exclusion criteria  Has a score of ≥4 when the weights from  the 5 criteria items are summed |  |
|  | Exclusion criteria:  Past head and neck radiation treatment, hepatitis C infection, AIDS, pre-existing  lymphoma, sarcoidosis, graft-versus-host disease, use of anticholinergic drugs (within a time shorter than fourfold the half-life of the drug) | Exclusion criteria: history of head and neck radiation treatment, active hepatitis C infection (with confirmation by PCR), AIDS, sarcoidosis, amyloidosis, graft-versus-host disease, and IgG4-related disease |  |

Supplemental Table S1 Classification criteria used in primary Sjögren’s syndrome. AECG American-European Consensus Group; ACR, American College of Rheumatology; AIDS, acquired immunodeficiency syndrome; ESSDAI, EULAR Sjögren’s syndrome Disease Activity Index; EULAR, European Alliance of Associations for Rheumatology; IgG, Immunoglobulin G; PCR, polymerase chain reaction; anti-SSA, anti-Sjögren's-syndrome-related antigen A; anti-SSB, anti-Sjögren's-syndrome-related antigen B

Classification in systemic lupus erythematosus

| **ACR 1997 criteria for SLE** | **EULAR/ACR 2019 SLE Classification Criteria** | | | |
| --- | --- | --- | --- | --- |
| Malar rash | Clinical domains |  | Immunologic domains |  |
| Discoid rash | **Constitutional domain**  Fever | 2 | **APS antibody domain**  Anticardiolipin IgG > 40 GPL OR  Anti-beta2GP1 IgG > 40 units OR  Lupus anticoagulant | 2 |
| Photosensitivity | **Cutaneous domain**  Non-scarring alopecia  Oral ulcers  Subacute cutaneous /discoid  Acute cutaneous lupus | 2  2  4  6 | **Complement protein domain**  Low C3 or low C4  Low C3 and low C4 | 3  4 |
| Oral ulcers | **Arthritis domain**  Synovitis or tenderness in at least 2 joints | 6 | **Highly specific antibodies** **domain**  Anti-dsDNA  Anti-Sm | 6  6 |
| Arthritis | **Neurological domain**  Delirium  Psychosis  Seizure | 2  3  5 |  |  |
| Serositis | **Serositis domain**  Pleural/ pericardial effusion  Acute pericarditis | 5  6 |  |  |
| Renal disorder | **Haematologic domain**  Leukopenia  Thrombocytopenia  Autoimmune haemolysis | 3  4  4 |  |  |
| Neurologic disorder | **Renal domain**  Proteinuria >0.5g/ 24 hours  Class II or V lupus nephritis  Class III or IV lupus nephritis | 4  8  10 |  |  |
| Haematologic disorder |  |  |  |  |
| Immunologic disorder |  |  |  |  |
| Antinuclear antibodies (ANA) |  |  |  |  |
| Requirement: ≥4 criteria | Requirement:  All patients classified as having SLE must have ANA ≥ 1:80  Patients must have ≥ 10 points to be classified as SLE  Only the highest criterion in a given domain counts  SLE classification requires points from at least one clinical domain | | | |

Supplemental Table S2 - Classification in systemic lupus erythematosus. ACR, American College of Rheumatology; ANA, anti-nuclear antibody; anti-dsDNA, anti-double stranded DNA; EULAR, European Alliance of Associations for Rheumatology; IgG, Immunoglobulin G; SLICC, the systemic lupus international collaborating clinic; anti-Sm, anti-Smith

Classification criteria for systemic sclerosis

| **ARA 1980 criteria for SSc** | **2013 ACR/EULAR criteria for SSc** |  |
| --- | --- | --- |
| **Major criterion** |  |  |
| Proximal cutaneous sclerosis/skin thickening (non‐ pitting) proximal to the MCPs, affecting other parts of the body | Skin thickening of the fingers of both hands extending proximal to the MCP joints | 9 |
| **Minor criterion** | **Skin thickening of the fingers^a^**  Puffy fingers  Sclerodactyly of the fingers (distal to the MCPs but proximal to the PIPs) | 2  4 |
| Sclerodactyly | **Fingertip lesions^a^**  Digital tip ulcers  Fingertip pitting scars | 2  3 |
| Digital pitting scars | Telangiectasia | 2 |
| Bibasilar pulmonary fibrosis | Abnormal nailfold capillaries | 2 |
|  | Pulmonary artery hypertension and/ or ILD^b^ | 2 |
|  | RP | 3 |
|  | SSc-related auto-antibodies (anti-centromere, anti-topomerase I (ant-Scl70), anti-RNApolyermase III | 3 |
| One major or ≥2 minor criteria | The total score is determined by adding the maximum weight (score) in each category. Patients with a total score of 9 are classified as having definite SSc | |

Supplemental Table S3 Classification criteria for systemic sclerosis. ^a^ only count the higher score, ^b^ max score is 2. ARA, American Rheumatism Association; MCP, metacarpophalangeal joints; RP, Raynaud’s phenomenon; SSc, systemic sclerosis

Classification criteria for idiopathic inflammatory myopathies

| Bohan and Peter 1975 | ACR/ EULAR 2017 | | |
| --- | --- | --- | --- |
| A) Symmetric proximal muscle weakness determined by physical examination | Variable | Score without muscle biopsy | Score with muscle biopsy |
| B) Elevation of serum skeletal muscle enzymes, including creatine kinase, aldolase, serum glutamate oxaloacetate and pyruvate transaminases, lactate dehydrogenase | Age of onset of first symptom ≥ 18 years and < 40 years  Age of onset of first symptom ≥ 40 years | 1.3  2.1 | 1.5  2.2 |
| C) The electromyographic triad of short, small, polyphasic motor unit potentials; fibrillations, positive sharp waves, and insertional irritability; and bizarre, high-frequency repetitive discharges | Objective symmetric weakness, usually progressive, of the proximal upper extremities | 0.7 | 0.7 |
| D) Muscle biopsy abnormalities of degeneration, regeneration, necrosis, phagocytosis, and an interstitial mononuclear infiltrate | Objective symmetric weakness, usually progressive, of the proximal lower extremities | 0.8 | 0.5 |
| E) Typical skin rash of DM, including a heliotrope rash or Gottron’s sign/papules | Neck flexors are relatively weaker than neck extensors | 1.9 | 1.6 |
|  | In the legs proximal muscles are relatively weaker than distal muscles | 0.9 | 1.2 |
|  | Heliotrope rash | 3.1 | 3.2 |
|  | Gottron´s papules | 2.1 | 2.7 |
|  | Gottron’s sign | 3.3 | 3.7 |
|  | Dysphagia or esophageal dysmotility | 0.7 | 0.6 |
|  | Anti-Jo-1 (anti-histidyl-tRNA synthetase) autoantibody present | 3.9 | 3.8 |
|  | Elevated serum levels of CK or LDH or AST or ALT | 1.3 | 1.4 |
|  | Muscle biopsy findings: | | |
|  | Endomysial infiltration of mononuclear cells surrounding, but not invading, myofibres |  | 1.7 |
|  | Perimysial and/or perivascular infiltration of mononuclear cells |  | 1.2 |
|  | Perifascicular atrophy |  | 1.9 |
|  | Rimmed vacuoles |  | 3.1 |
| Polymyositis:   1. Definite - All of A-D 2. Probable – any 3 of A-D 3. Possible – any 2 of A-D | Results range (total score):  Do not meet criteria for IIM  Possible IIM  Probable IIM  Definite IIM | <5.3  5.3-5.49  5.5-7.49  ≥7.5 | <6.5  6.5-6.69  6.7-8.69  ≥8.7 |
| Dermatomyositis:   1. Definite – E and 3 of A-D 2. Probable – E plus 2 of A-D 3. Possible – E and 1 of A-D |  |  |  |

Supplemental Table S4 Classification criteria for IIM. CK, creatine kinase; LDH, lactate dehydrogenase; AST, aspartate aminotransferase; ALT, alanine aminotransferase

**Identification of studies via databases and registers**

Records identified:

(n = 1916)

**Identification**

Records excluded using automation tools:

Not interventional (n=501)

Not phase III (n = 1155)

Not completed (n=135)

Duplicates (n=2)

Records screened

(n = 1916)

Reports not retrieved

(n = 0)

Reports sought for retrieval

(n = 123)

**Screening**

Reports excluded:

Long term extension (n = 7)

Open label (n=23)

Wrong disease (n = 3)

Wrong therapy type (n = 43)

Phase-II study (n=2)

Not core trial (n=3)

Reports assessed for eligibility

(n = 123)

Studies included in review

(n = 42)

**Included**

Supplemental Figure S5 – PRISMA diagram outlining the search strategy for phase-III clinical trials in connective tissue diseases. Records were identified from clinicaltrials.gov, screened initially using automation tools, then manually assessed for eligibility by two authors (SD and AM).

| NCT ID | Investigational drug/ trial name | Completion date | Inclusion | Exclusion |
| --- | --- | --- | --- | --- |
| IIM |  |  |  |  |
| NCT00335985 | GB-0998 | 2009 | Definite PM or DM according to the criteria of Bohan and Peter (1975) |  |
| NCT01165008 | Anakinra | 2010 | Diagnosis of PM, DM or IBM based Peter and Bohan's and Grigg's criteria |  |
| NCT02728752 | Octagam 10% | 2021 | Definite or probable DM according to Bohan and Peter criteria | Overlap myositis (except for overlap with Sjögren's syndrome), connective tissue disease associated DM, IBM, PM, juvenile DM or drug-induced myopathy |
| NCT03813160 | Lenabasum (DETERMINE) | 2021 | Definite or probable DM diagnosed according to Bohan and Peter, or ACR/EULAR criteria 2017 for DM | Overlap with features of SSc, SLE, Sjögren’s syndrome or rheumatoid arthritis is allowed if the dominant clinical disease is DM |
| Primary Sjögren’s |  |  |  |  |
| NCT00632866 | Hydroxychloroquine (JOQUER) | 2012 | Primary Sjögren’s according to the AECG criteria 2002 | Sjögren’s syndrome associated with other autoimmune diseases |
| NCT00740948 | Rituximab (TEARS) | 2013 | Primary Sjögren’s according to the AECG criteria 2002 | Secondary Sjögren’s syndrome |
| NCT01601028 | Hydroxychloroquine | 2013 | Primary Sjögren’s according to the AECG criteria 2002 |  |
| NCT01782235 | Tocilizumab (ETAP) | 2018 | Primary Sjögren’s according to the AECG criteria 2002 | Systemic auto-immune disease |
| NCT02067910 | Abatacept SC (ASAPIII) | 2019 | Primary Sjögren’s according to the AECG criteria 2002 | Presence of any other connective tissue disease |
| NCT02915159 | Abatacept | 2019 | 2016 ACR/EULAR Classification Criteria for Sjögren's Syndrome | Secondary Sjögren’s syndrome, other medical condition associated with sicca syndrome, another systemic autoimmune disease, inflammatory conditions, severe fibromyalgia or other medical conditions |
| SLE |  |  |  |  |
| NCT00470522 | Methotrexate (SMILE) | 2001 | Diagnosis of SLE |  |
| NCT00035308 | Abetimus sodium (LJP394) | 2002 | ACR SLE criteria 1982 |  |
| NCT00137969 | Rituximab (EXPLORER) | 2008 | ACR SLE criteria 1997 | Significant, uncontrolled medical disease in any organ system not related to SLE that in the investigator's opinion would preclude subject participation |
| NCT00410384 | Belimumab (BLISS-76) | 2010 | ACR SLE criteria 1997 | Clinical evidence of significant unstable or uncontrolled acute or chronic diseases not due to SLE (i.e. cardiovascular, pulmonary, hematologic, gastrointestinal, hepatic, renal, neurological, malignancy or infectious diseases) which, in the opinion of the principal investigator, could confound the results of the study or put the subject at undue risk |
| NCT00424476 | Belimumab (BLISS-52) | 2010 | ACR SLE criteria 1997 | Clinical evidence of significant unstable or uncontrolled acute or chronic diseases not due to SLE (ie, cardiovascular, pulmonary, hematologic, gastrointestinal, hepatic, renal, neurological, malignancy or infectious diseases) which, in the opinion of the principal investigator, could confound the results of the study or put the subject at undue risk |
| NCT00624338 | Atacicept (APRIL/SLE) | 2012 | ACR SLE criteria 1997 |  |
| NCT00282347 | Rituximab (LUNAR) | 2013 | ACR SLE criteria 1997 | Significant or uncontrolled medical disease in any organ system not related to SLE or LN, which, in the investigator's opinion, would preclude subject participation |
| NCT01196091 | Tabalumab (ILLUMINATE 1) | 2015 | ACR SLE criteria 1997 | Any condition or event that, in the investigator’s opinion, would pose an unacceptable risk to the patient were excluded |
| NCT01205438 | Tabalumab (ILLUMINATE 2) | 2015 | ACR SLE criteria 1997 | Any condition or event that, in the investigator’s opinion, would pose an unacceptable risk to the patient were excluded |
| NCT01261793 | Epratuzumab (EMBODY 2) | 2015 | ACR SLE criteria 1982 (not including neurological criteria) |  |
| NCT01262365 | Epratuzumab (EMBODY 1) | 2015 | ACR SLE criteria 1982 (not including neurological criteria) |  |
| NCT01395745 | Blisibimod (CHABLIS SC1) | 2016 | ACR SLE criteria 1997 |  |
| NCT02446899 | Anifrolumab (TULIP 2) | 2018 | ACR SLE criteria 1997 | Diagnosis (within 1 year) of MCTD or any history of overlap syndromes of SLE or SSc. A history of, or current, inflammatory joint or skin disease other than SLE. History of, or current, inflammatory joint or skin disease other than SLE that, in the opinion of the Investigator, could interfere with the inflammatory arthritis or skin assessments and confound the disease activity assessments |
| NCT02446912 | Anifrolumab (TULIP 1) | 2018 | ACR SLE criteria 1997 | Diagnosis (within 1 year) of MCTD or any history of overlap syndromes of SLE or SSc. A history of, or current, inflammatory joint or skin disease other than SLE. History of, or current, inflammatory joint or skin disease other than SLE that, in the opinion of the Investigator, could interfere with the inflammatory arthritis or skin assessments and confound the disease activity assessments |
| NCT02504645 | IPP-201101 (LUPUZOR) | 2018 | ACR SLE criteria 1997 | Any concomitant medical condition unrelated to SLE that may interfere with his or her safety or with evaluation of the study drug, as determined by the investigator |
| NCT03021499 | Voclosporin (AURORA) | 2019 | ACR SLE criteria 1997 | Any overlapping autoimmune condition for which the condition or the treatment of the condition may affect the study assessments or outcomes (e.g. SSc with significant pulmonary hypertension; any condition for which additional immunosuppression is indicated). Overlapping conditions for which the condition or treatment is not expected to affect assessments or outcomes (e.g., Sjögren’s syndrome) are not excluded |
| NCT01639339 | Belimumab (BLISS-LN) | 2020 | ACR SLE criteria 1997 | Clinical evidence of significant unstable or uncontrolled acute or chronic diseases not due to SLE (ie, cardiovascular, pulmonary, hematologic, gastrointestinal, hepatic, renal, neurological, malignancy, or infectious diseases) which, in the opinion of the principal investigator, could confound the results of the study or put the subject at undue risk |
| NCT03312907 | Rituximab/ Belimumab (BLISS-BELIEVE) | 2021 | ACR SLE criteria | Clinical evidence of significant unstable or uncontrolled acute or chronic diseases not due to SLE |
| NCT03616964 | Baricitinib (BRAVE II) | 2021 | ACR SLE criteria 1997 | Have clinical evidence of significant unstable or uncontrolled acute or chronic diseases not due to SLE (ie, cardiovascular, pulmonary, hematologic, gastrointestinal, hepatic, renal, neurological, malignancy or infectious diseases) which, in the opinion of the principal investigator, could confound the results of the study or put the subject at undue risk |
| NCT00429377 | Tacrolimus | 2005 | ACR SLE criteria 1982 | Patients unsuitable for other reasons according to the judgment of the attending physician |
| SSc |  |  |  |  |
| NCT00704665 | Relaxin | 2001 | Diffuse SSc |  |
| NCT00070590 | Bosentan | 2005 | Diffuse or limited SSc | Interstitial Lung Disease due to other conditions than SSc |
| NCT00348296 | High-dose IVIG (Venoglobulin-IH) | 2009 | diffuse SSc |  |
| NCT00004563 | Cyclophosphamide (SLS) | 2013 | ACR preliminary criteria for the classification of SSc 1980 |  |
| NCT01553981 | Tadalafil | 2014 | ACR preliminary criteria for the classification of SSc 1980 |  |
| NCT01532869 | Tocilizumab | 2016 | ACR preliminary criteria for the classification of SSc 1980 | Rheumatic autoimmune disease other than SSc |
| NCT01748084 | Rituximab (RECOVER) | 2016 | SSc fulfilling ACR 1980 or LeRoy's criteria 1988 | Overlap syndrome defined by clinical symptoms and positive specific auto-antibodies (anti-CCP, anti-SSA, anti-dsDNA anti-Sm) |
| NCT02896205 | Mycophenolate (MYILD) | 2017 | ACR preliminary criteria for the classification of SSc 1980 | Inflammatory myositis, overlap syndrome or MCTD |
| NCT01570764 | Cyclophosphamide (SCLEROCYC) | 2018 | ACR criteria for SSc 1980 and/or Leroy and Medsger 2001 diagnostics criteria | Association to another connective disease: SLE, syndrome of Gougerot-Sjögren with anti-SSA/SSB, MCTD |
| NCT02597933 | Nintedanib | 2018 | 2013 ACR/ EULAR criteria SSc |  |
| NCT02453256 | Tocilizumab (focuSSced) | 2019 | 2013 ACR/ EULAR criteria SSc | Rheumatic autoimmune disease other than SSc |
| NCT04274257 | Rituximab (DesiReS) | 2020 | 2013 ACR/ EULAR criteria SSc |  |

Supplemental Table S6 Classification criteria used in clinical trials. American college of rheumatology, ACR; European-American consensus group, AECG; American rheumatology association, ARA; dermatomyositis, DM; European League Against Rheumatism, EULAR; inclusion body myositis, IBM; MCTD, mixed connective tissue disease; polymyositis, PM; systemic lupus erythematosus ,SLE; systemic sclerosis, SSc; intravenous immunoglobulin, IVIG; anti-cyclic citrullinated peptide; anti-CCP, anti-double stranded DNA, anti-dsDNA; anti-Smith, anti-Sm; anti-Sjögren’s Syndrome A, anti-SSA; Anti-Sjögren’s Syndrome B, anti-SSB


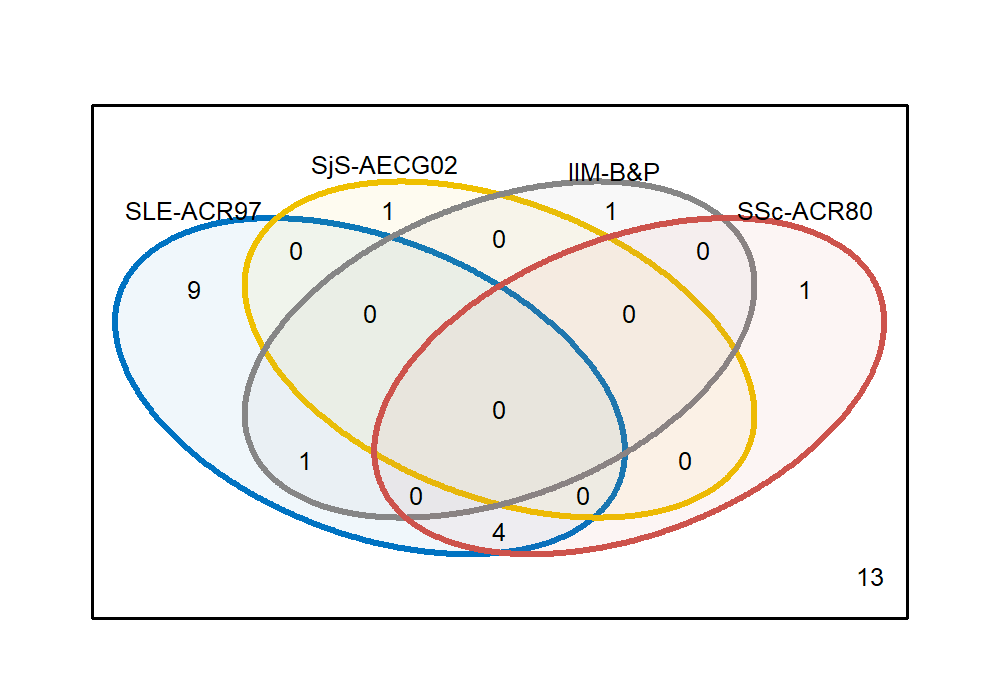


Supplemental Figure S7 - classification criteria utilised in Phase-III clinical trials, applied to patients with a diagnosis of MCTD


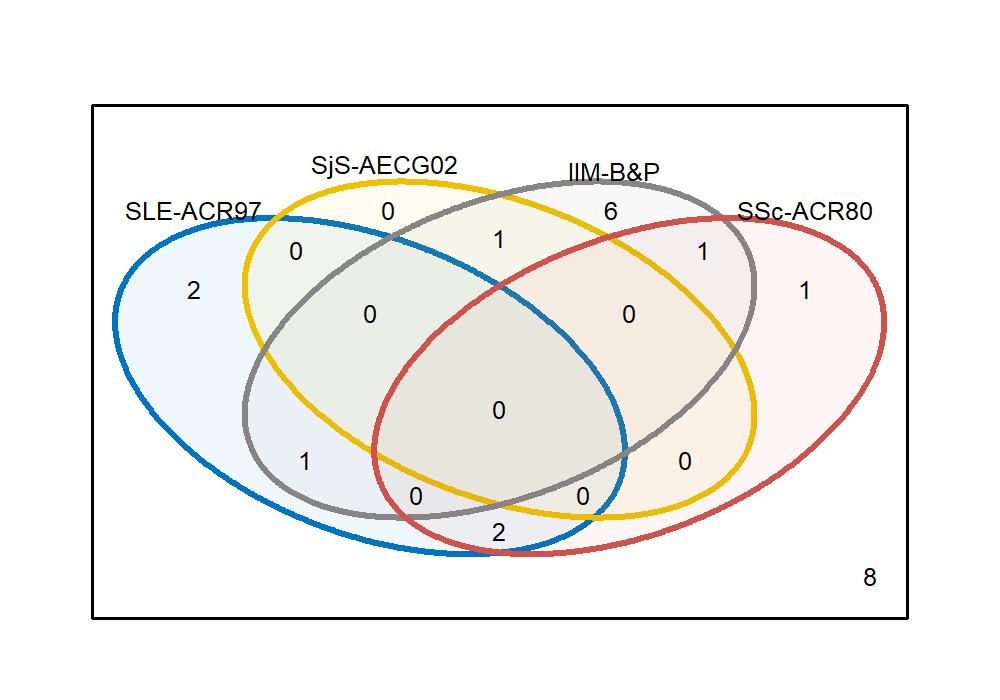


Supplemental Figure S8 – classification criteria utilised in Phase-III clinical trials, applied to patients with a diagnosis of myositis


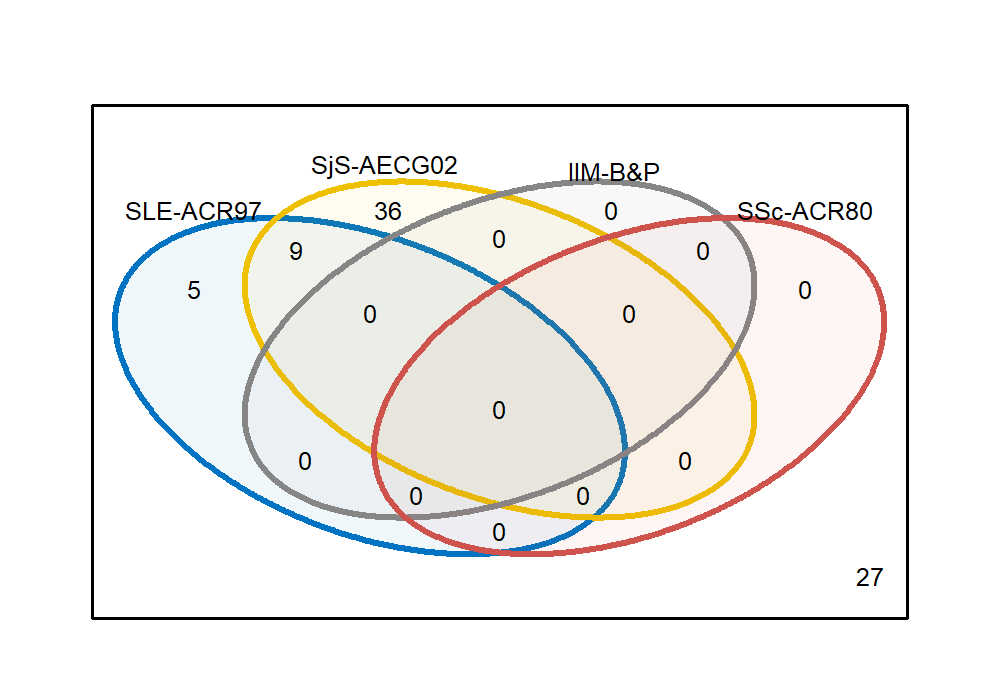


Supplemental Figure S9 - classification criteria utilised in Phase-III clinical trials, applied to patients with a diagnosis of primary Sjögren’s syndrome


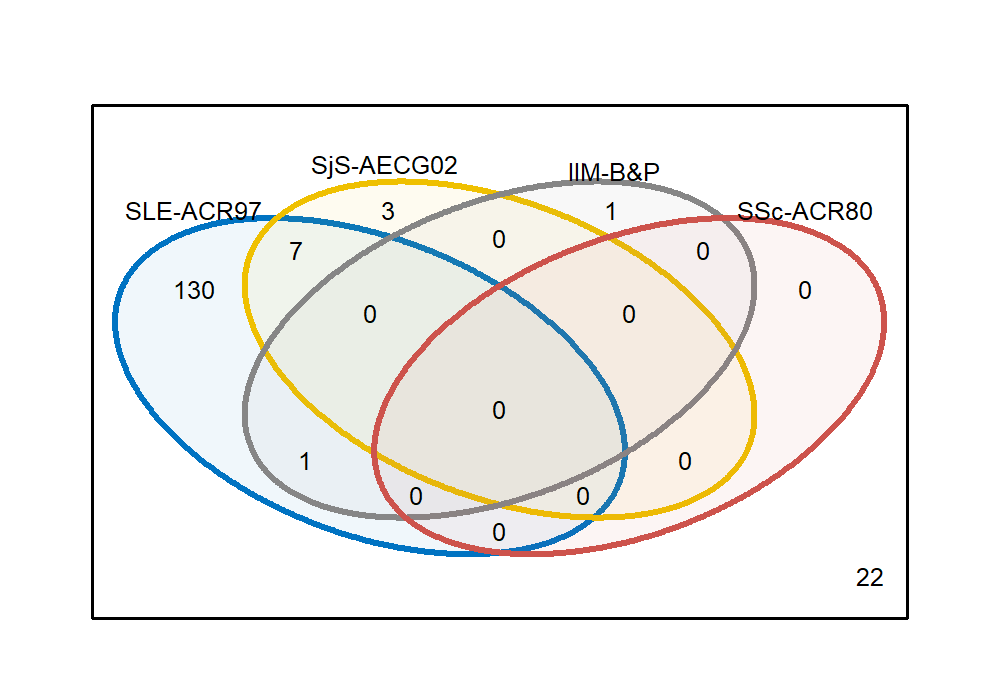


Supplemental Figure S10 - classification criteria utilised in Phase-III clinical trials, applied to patients with a diagnosis of systemic lupus erythematosus


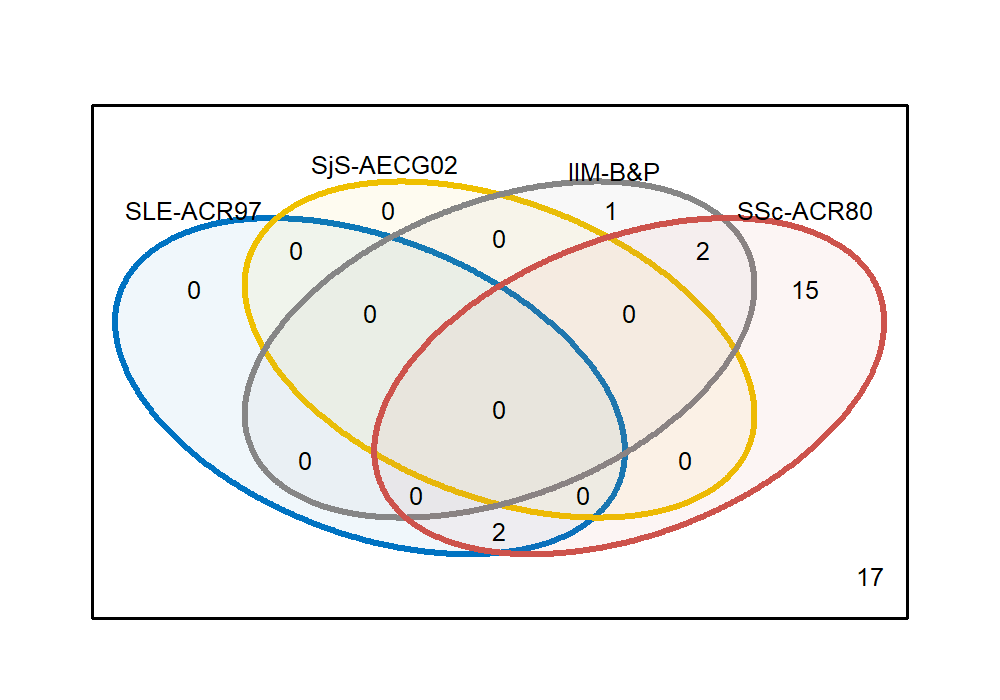


Supplemental Figure S11 - classification criteria utilised in Phase-III clinical trials, applied to patients with a diagnosis of systemic sclerosis


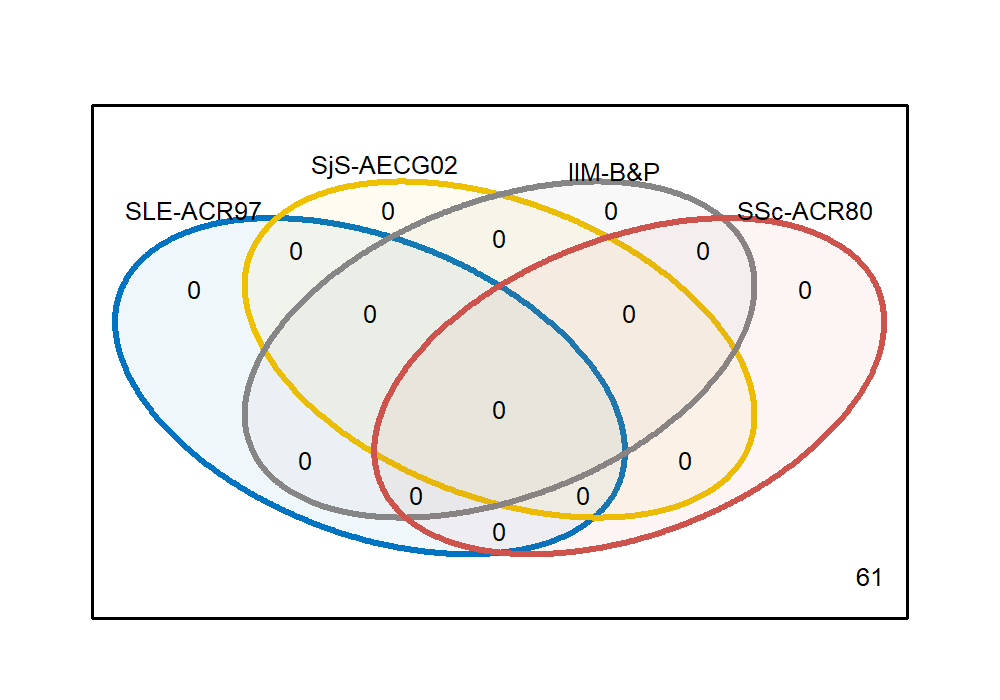


Supplemental Figure S12 - classification criteria utilised in Phase-III clinical trials, applied to patients with a diagnosis of undifferentiated connective tissue disease
